# A Principled Account of AMR Global Governance Solidarity, Subsidiarity, and Stewardship

**DOI:** 10.1007/s10728-023-00456-w

**Published:** 2023-02-25

**Authors:** Thana C. de Campos-Rudinsky

**Affiliations:** School of Government, Pontificia Universidad Catolica de Chile, Santiago, Chile

**Keywords:** Antimicrobial resistance, Global health governance, Solidarity, Subsidiarity, Stewardship

## Abstract

This commentary defines what shared yet differentiated ethical responsibilities to tackle antimicrobial resistance (AMR) mean, by introducing a threefold principled account of AMR global governance. It argues that the principles of solidarity, subsidiarity, and stewardship can be especially helpful for further justifying some of the universal, differentiated, and individual responsibilities that Van Katwyk et al propose. The upshot of my threefold principled account of AMR global governance is a less ambitious AMR treaty, one that can only justify (i) universal duties of global coordination (as per the principle of solidarity); (ii) differentiated duties to local communities, which bear the primary AMR responsibilities (as per the principle of subsidiarity); and (iii) individualized duties for ensuring truthful, evidence-based, consistent, and timely shared accountable communication (as per the principle of stewardship).

Global health experts have widely debated the need for an effective global governance structure to regulate the global collective problem of antimicrobial resistance (AMR) [1–3]. An effective regulatory AMR system would need to coordinate collective action not only across countries but also across sectors (e.g., human health, animal, agricultural, and environmental), while also taking into consideration justice-related questions of access to life-saving antimicrobials in developing countries, balanced against innovation-related questions on the R&D of new antimicrobials, coupled with management-related questions pertaining to antimicrobial overuse in some other countries [1–3]. AMR indeed poses a complex coordination problem that justifies – and in fact requires – the interference of the law to be properly solved in a way that upholds the common good. In this vein, an AMR treaty has been argued for as the most effective legal framework to tackle the AMR problem [2, 3]. Among other things, such AMR treaty would establish how to allocate responsibilities among different AMR stakeholders, across countries, and across sectors. These are shared yet differentiated responsibilities. More specifically, as Van Katwyk et al. [2] have argued, these would include universal, differentiated, and individual responsibilities.

Building on Van Katwyk et al.’s model [2], my commentary further defines these shared yet differentiated ethical responsibilities to tackle AMR by introducing a threefold principled account of AMR global governance. I argue that the principles of solidarity, subsidiarity, and stewardship can be especially helpful for further justifying some of the universal, differentiated, and individual responsibilities that Van Katwyk et al. [2] propose. My threefold principled account of AMR global governance is helpful for designing a *good* global governance structure for AMR regulation. By ‘good’ I mean a global governance structure that is ethical and effective in upholding the common good. By ‘common good’ I do not mean the maximized welfare for the greatest number. Rather, I mean the set of values and reasons that justify collaboration with others in a way that enables mutual flourishing. My proposed threefold principled account of AMR global governance provides the practical reasons that justify why some of the treaty provisions that Van Katwyk et al. [2] suggest are necessary for upholding the common good, while others may lack a justification that goes beyond a utilitarian cost-benefit analysis.

## Universal Responsibilities and the Principle of Solidarity

The proposed AMR treaty establishes universal duties of global coordination, including for example, setting a global target and benchmark of progress, organizing an annual multi-stakeholder forum coupled with an independent scientific stock-take to inform policy decisions on AMR [2, 3]. I would suggest that the principle of solidarity can provide further justification for these universally-shared commitments. Solidarity is a fundamental principle of public international law in general, and of the international law of human rights in particular [4, 5]. More recently, the principle of solidarity has also been discussed in the context of bioethics and public health ethics [1, 5–10]. As a principle of justice, solidarity has the purpose of protecting the human dignity of each human life individually, in the reality of our mutual vulnerabilities and interconnectedness [4, 5]. The principle of solidarity therefore entails, in the context of global health justice, a shared commitment, among all global health stakeholders to uphold the good and the flourishing of each individual in every community [5].

The universally-shared responsibilities that the AMR treaty contains are predicated on the principle of solidarity because they are grounded on our shared vulnerability to the problem of the reduction of antimicrobial effectiveness [1]. This is a global common pool resource challenge that needs to be collectively managed [2, 3]. It is our shared vulnerability to this collective problem that justifies our shared commitment for a coordinated response among persons, institutions across sectors, and nations. Van Katwyk et al.’s model [2] of universal responsibilities dovetails with the principle of solidarity. However, their model also rightly calls for “an ethically fair AMR agreement [that] would feature different targets and obligations for different countries” [2]. The authors here establish two main criteria for the allocation of different responsibilities for different AMR stakeholders: namely, ‘contribution to the problem (e.g., historical rates of antimicrobial use) and capacity to respond to it (e.g., economic states)’ [2].

It is not clear, however, whether these two criteria are equally relevant in determining obligations or whether one criterion takes precedence. Stipulating contribution is typically relevant for establishing the legal causal link between the problem and those who, having caused it, have a legal duty to remedy it. Contribution is therefore key if the purpose of the AMR treaty is to assign legal responsibilities for addressing the AMR problem. However, if the AMR problem goes beyond what is legally required and calls also for more general, ethical responsibilities – as I think it does -- then the broader criterion of capacity should be the chief one for allocating differentiated responsibilities. My second principle of AMR global governance explains why and how.

## Differentiated Responsibilities and the Principle of Subsidiarity

As a structural principle, subsidiarity justifies a bottom-up approach to decisional authority allocation among multi-level stakeholders [5, 6, 8]. Accordingly, subsidiarity justifies allocating responsibilities firstly to locals [5, 6, 8]. The reason is that local stakeholders typically have better knowledge of the epidemiological reality and the medical culture in a said country, being therefore *prima facie* best positioned to effectively solve problems requiring the coordination of persons, institutions across sectors, and nations. The principle of subsidiary justifies delegating to higher levels of governance only as necessary, when the local capacity to solve a problem has proven insufficient [5, 6, 8]. In short, to apply the principle of subsidiarity to AMR global governance means to assign to local individuals and institutions the primary responsibility to manage and coordinate AMR’s health culture.

The principle of subsidiarity and its emphasis on local communities’ primary responsibility may challenge Van Katwyk et al.’s model, according to which high income countries (HIC) ‘have a greater moral obligation to shoulder greater burdens when it comes to tackling the resulting global fallout of AMR’ [2]. One way in which HICs’ greater moral responsibility would be discharged in Van Katwyk et al.’s model would be, for example, by requiring HICs to donate greater amounts to an international AMR fund [2]. Presumably, Van Katwyk et al. call for greater responsibility falling on HICs based on the criterion of contribution. Although this is not explicitly mentioned, it can be inferred because HICs have historically overused antimicrobials and have therefore arguably contributed more to the global burden of AMR [2]. Quite possibly, however, their call for greater responsibility falling on HICs is also based on the criterion of capacity, given that HICs have more economic capacity to implement AMR solutions [2]. If this is so, there are two main challenges to Van Katwyk et al.’s claim.

First, the fact that a certain group of powerful nations has contributed more to the global burden of AMR does not automatically give such a group license to interfere—for example, through an international AMR fund—in developing countries’ domestic affairs and in how they should manage their AMR issues. This is not to say that all AMR treaty provisions should be banished because they all interfere with domestic affairs. There is a relevant moral difference between (i) an international AMR fund that dictates how locals should micro-manage their AMR problems, and (ii) an AMR treaty where parties establish together a global target and benchmark of global progress, and multilaterally agree to an annual forum and an independent scientific stock-take to help inform AMR policies [2, 3]. As a collaboration and coordination response that all countries technically agree with and do together, an AMR fund should obviously not require HICs to micromanage activities in LICs, unless LICs invite such intervention on the basis, for example, of need or lesser capacity. While this is what the international law of treaties would determine, the coloniality of global health has also been a reality that Van Katwyk et al.’s proposal should not neglect. The principle of subsidiarity addresses the coloniality problem by necessitating a bottom-up approach.

Although Van Katwyk et al.’s model does not explicitly adopt a top-down global governance approach, it leaves room for this kind of top-down measure (e.g., an international AMR fund potentially micro-managing local AMR problems) that would need further justification to be reasonably accepted. For example, if certain top-down interferences in local affairs were more efficient and cost-effective than leaving local governments to first try to manage their AMR problems by themselves, then it seems that Van Katwyk et al.’s model, predicated on a utilitarian cost-benefit analysis, would favor such interferences. However, if one were to follow the ethical reasons that the principle of subsidiarity provides, one would object to them, for the purpose of taking inclusion and participation of local communities seriously. Subsidiarity would tolerate certain inefficiencies for the sake of respecting local communities’ right to address their own AMR issues prior to asking for other countries’ assistance.

Secondly, the fact that a certain group of powerful nations has more greatly contributed to the global burden of AMR does not automatically entail that such group also possesses greater capacity effectively and ethically to address the AMR global problem. Likewise, the fact that a certain group of powerful nations has more financial capacity to address AMR problems does not automatically entail that such group also possesses greater AMR stewardship capacity. On the contrary, local communities have *prima facie* and *ceteris paribus* greater stewardship capacity, given their better knowledge of the epidemiological reality and the medical culture in their community. This leads me to the third principle of AMR global governance.

## Individual Responsibilities and the Principle of Stewardship

As a governing principle, stewardship justifies when intervening and assisting are reasonable and legitimate acts of care and when not, further complementing and specifying the principles of solidarity and subsidiarity [8]. The principle of stewardship in general and its particular application to AMR have been critically discussed in the bioethics and public health ethics literature [1, 8, 11, 12]. The steward has a specific duty of care in its mandate [8]. Truthful and accountable communication between the steward and those under their leadership is the central component of their mandate [8].

Van Katwyk et al. claim that “one of the greatest hurdles for global antimicrobial stewardship has been the lack of internationally standardized and transparent data collection on antimicrobial usage and AMR” [2]. I would suggest that another way of putting this is: there is a lack of universally common AMR language – i.e., internationally standardized usage definitions, measurement methods, and data processing. And the lack of a universally common AMR language leads to the lack of truthful, evidence-based, consistent, and timely shared communication. This is precisely where the principle of stewardship can offer guidance.

In discharging the AMR mandate to conserve antibiotic effectiveness, the principle of stewardship calls for the virtues of clarity and prudence in striving to share truthful and accountable communication with other stakeholders. The AMR treaty entrusts this mandate to all parties to the treaty. In doing so, parties become stewards of the AMR mandate. The virtue of clarity would require stewards to focus on honing their AMR communication skills with one another. The virtue of prudence would require stewards to move with caution, by limiting their mandates to fulfilling their mission of sharing truthful and accountable communications necessary to conserve antibiotic effectiveness. The principle of stewardship therefore would justify a less ambitious AMR treaty -- one that channels global health’s and AMR’s limited resources towards sharing truthful and accountable communications. This should be AMR mandate’s priority in conserving antibiotic effectiveness. Under my proposed account of AMR global governance, any treaty provisions that go beyond this priority would need to be further justified.

## Conclusion

I have defined what shared yet differentiated ethical responsibilities to tackle AMR mean, by introducing a threefold principled account of AMR global governance. I argued that the principles of solidarity, subsidiarity, and stewardship can be especially helpful for further justifying some of the universal, differentiated, and individual responsibilities that Van Katwyk et al. propose [2]. The upshot of my account of AMR global governance would be a less ambitious AMR treaty, one that can only justify (i) universal duties of global coordination (as per the principle of solidarity); (ii) differentiated duties to local communities, which bear the primary AMR responsibilities (as per the principle of subsidiarity); and (iii) individualized duties for ensuring truthful, evidence-based, consistent, and timely shared accountable communication (as per the principle of stewardship).
